# Adjusting plasma or serum zinc concentrations for inflammation: Biomarkers Reflecting Inflammation and Nutritional Determinants of Anemia (BRINDA) project

**DOI:** 10.1093/ajcn/nqz304

**Published:** 2020-04-08

**Authors:** Christine M McDonald, Parminder S Suchdev, Nancy F Krebs, Sonja Y Hess, K Ryan Wessells, Sanober Ismaily, Sabuktagin Rahman, Frank T Wieringa, Anne M Williams, Kenneth H Brown, Janet C King

**Affiliations:** 1 Children's Hospital Oakland Research Institute, Oakland, CA, USA; 2 Department of Pediatrics, School of Medicine, University of California, San Francisco, CA, USA; 3 Rollins School of Public Health, Emory University, Atlanta, GA, USA; 4 Nutrition Branch, CDC, Atlanta, GA, USA; 5 Section of Nutrition, Department of Pediatrics, University of Colorado School of Medicine, Aurora, CO, USA; 6 Department of Nutrition, University of California, Davis, CA, USA; 7 International Centre for Diarrhoeal Disease Research, Bangladesh (icddr,b), Dhaka, Bangladesh; 8 School of Medicine, Griffith University, Gold Coast, Queensland, Australia; 9 Research Institute for Development, Montpellier, France; 10 McKing Consulting Corporation, Atlanta, GA, USA

**Keywords:** zinc, inflammation, micronutrients, nutritional assessment, undernutrition

## Abstract

**Background:**

The accurate estimation of zinc deficiency at the population level is important, as it guides the design, targeting, and evaluation of nutrition interventions. Plasma or serum zinc concentration (PZC) is recommended to estimate zinc nutritional status; however, concentrations may decrease in the presence of inflammation.

**Objectives:**

We aimed to assess the relation between PZC and inflammation in preschool children (PSC; 6–59 mo) and nonpregnant women of reproductive age (WRA; 15–49 y), and to compare different inflammation adjustment approaches, if adjustment is warranted.

**Methods:**

Cross-sectional data from 13 nationally representative surveys (18,859 PSC, 22,695 WRA) from the Biomarkers Reflecting Inflammation and Nutritional Determinants of Anemia (BRINDA) project were analyzed. Correlation and decile analyses were conducted, and the following 3 adjustment methods were compared if a consistent negative association between PZC and C-reactive protein (CRP) or α-1-acid glycoprotein (AGP) was observed: *1*) exclude individuals with CRP > 5 mg/L or AGP > 1 g/L; *2*) apply arithmetic correction factors; and *3*) use the BRINDA regression correction (RC) approach.

**Results:**

In 6 of 12 PSC surveys, the estimated prevalence of zinc deficiency increased with increasing CRP deciles, and to a lesser extent, with increasing AGP deciles. In WRA, the association of PZC with CRP and AGP was weak and inconsistent. In the 6 PSC surveys in which adjustment methods were compared, application of RC reduced the estimated prevalence of zinc deficiency by a median of 11 (range: 4–18) percentage points, compared with the unadjusted prevalence.

**Conclusions:**

Relations between PZC and inflammatory markers were inconsistent, suggesting that correlation and decile analyses should be conducted before applying any inflammation adjustments. In populations of PSC that exhibit a significant negative association between PZC and CRP or AGP, application of the RC approach is supported. At this time, there is insufficient evidence to warrant inflammation adjustment in WRA.

## Introduction

At least 17% of the global population is at risk of inadequate zinc intake, making zinc deficiency one of the most prevalent micronutrient deficiencies worldwide (1). Zinc is an essential trace element that functions as a catalyst, structural element, or regulatory ion in many metabolic processes, including DNA transcription and gene expression, signal transduction pathways, and endocrine function (2). As such, zinc is critically important for immune function, reproductive health, child growth, and development. In 2011, it was estimated that ∼116,000 child deaths per year are attributable to zinc deficiency (3).

The ability to accurately estimate the prevalence of zinc deficiency at the population level is important to guide the design and implementation of strategies to reduce the burden of zinc deficiency. Although a “gold standard” biomarker does not currently exist for the accurate assessment of zinc nutrition, the Zinc Expert Panel of Biomarkers of Nutrition for Development recently recommended the use of plasma or serum zinc concentrations (PZC) along with dietary zinc intake, and height-for-age of growing infants and children for the measurement of zinc status (4). Furthermore, the International Zinc Nutrition Consultative Group (IZiNCG) recommends that when the prevalence of low PZC is >20%, the risk of zinc deficiency is considered elevated and should be addressed through public health nutrition interventions to improve zinc status (5).

One of the limitations of PZC as an indicator of zinc nutrition is that concentrations may be depressed in the presence of inflammation (6–11). The acute-phase response can lead to a redistribution of zinc from the plasma or serum to the liver (12). In settings with a high burden of infection, this could lead to elevated estimates of the prevalence of nutritional zinc deficiency. C-reactive protein (CRP) and α-1-acid glycoprotein (AGP) are 2 acute-phase proteins most commonly assessed to measure the acute-phase response. CRP rises quickly and acutely in response to infection, whereas AGP rises more gradually and is reflective of longer-term exposure to inflammation (13, 14). Several approaches have been evaluated to address the issue of inflammation in the interpretation of other biomarkers of micronutrient status in the Biomarkers Reflecting Inflammation and Nutritional Determinants of Anemia (BRINDA) project (15–20); however, they have not been compared in the context of PZC. The objectives of this study are *1*) to determine whether there is a need to adjust PZC for inflammation to estimate the prevalence of nutritional zinc deficiency in preschool children (PSC) and nonpregnant women of reproductive age (WRA); and, if adjustment is warranted, *2*) to determine whether it is necessary to adjust PZC for CRP, AGP, or both; and *3*) to compare the different adjustment approaches for correcting for inflammation and estimating the prevalence of zinc deficiency.

## Methods

We used cross-sectional survey data from the BRINDA project (www.brinda-nutrition.org). A description of the methods used to identify data sets, define inclusion and exclusion criteria, and manage the BRINDA database has been previously reported (15). Briefly, the data used for this analysis were from nationally representative surveys that *1*) were conducted after 2004; *2*) utilized representative sampling designs; *3*) had target groups including PSC (aged 6–59 mo), WRA (15–49 y), or both; *4*) measured PZC; and *5*) measured CRP, AGP, or both (**Supplemental Figure 1**). The application of these criteria yielded 9 data sets that included data from PSC and WRA, 3 additional data sets that included only PSC, and 1 additional data set that included only WRA. Of the 12 data sets with data from PSC, 6 measured both CRP and AGP, 4 measured CRP only, and 2 measured AGP only. Of the 10 data sets with data from WRA, 6 measured both CRP and AGP, and 4 measured CRP only. Only 2 data sets assessed malaria status: Malawi and Cameroon.

### Laboratory analysis

Venous blood was obtained from participants in all surveys except Mongolia, which obtained a combination of capillary and venous blood samples. PZCs were measured in plasma or serum in 4 and 8 of the data sets, respectively. Fasting status, time of blood draw, and time from blood draw to separation are important factors to consider in the interpretation of PZC (4, 21); however, as indicated in **Supplemental Table 1**, these variables were not consistently reported across data sets. Inductively coupled plasma optical emission spectrometry, inductively coupled plasma mass spectrometry, and atomic absorption spectroscopy were used to assess PZC in 4, 1, and 8 data sets, respectively. Laboratory analysis of PZC was performed in 8 different laboratories. CRP and AGP were measured using a variety of methods including sandwich ELISAs (22, immunoassays, turbidimetry, and nephelometry (**Supplemental Table 1**).

### Case definitions

Zinc deficiency was defined according to IZiNCG recommendations (4, 21). More specifically, in PSC, PZC cutoffs of <65 μg/dL and <57 μg/dL were used to define zinc deficiency depending on whether the blood sample was obtained during the morning or afternoon, respectively. In both instances, a nonfasting state was presumed. In WRA, a PZC cutoff of <70 μg/dL was used if the blood sample was drawn in the morning in a fasting state, <66 μg/dL was used if the blood sample was taken in the morning in a nonfasting state, and <59 μg/dL was used if the blood sample was drawn in the afternoon when a nonfasting state was presumed. Inflammation was defined according to a CRP concentration > 5 mg/L and/or an AGP concentration > 1 g/L (23). Malaria infection based on a rapid diagnostic kit was defined as either positive or negative.

### Statistical analysis

As aforementioned, we restricted the data sets to include observations where PZC and CRP or AGP (or both) were available. CRP concentrations > 40 mg/L were not included in the calculation of deciles, similarly to prior BRINDA methods (16, 17), but were retained for other analyses. We also excluded observations where the PZC was above the maximum value of 160.9 μg/dL reported in the 2013–2014 US NHANES (24), because these were considered to likely be biologically implausible values that reflected contamination. Descriptive statistics were calculated for each data set using SAS version 9.4 (SAS Institute). SAS SURVEY procedures were used to adjust for the cluster, strata, and sampling weight of each survey when calculating means, frequencies, and their corresponding 95% CIs, except in the case of Mongolia which did not apply a complex survey design. Initially, Spearman rank correlations between PZC, CRP, and AGP concentrations in each data set were calculated using the PROC RANK and PROC CORR procedures, accounting for the sampling weight in the latter. To further explore the association between PZC and inflammation, we used SAS SURVEYMEANS and SURVEYFREQ procedures to estimate mean PZC and prevalence of zinc deficiency by CRP decile and AGP decile in each data set. If the correlation and decile analyses indicated a significant negative association between PZC and either acute-phase protein, the following 3 adjustment approaches were applied and compared. We pursued country-specific analyses and decided not to pool the data in order to maintain the considerable heterogeneity across data sets.

#### Exclusion approach

The exclusion approach simply excluded observations in which either the CRP concentration was >5 mg/L, the AGP concentration was >1 g/L, or both. The mean PZC and the prevalence of zinc deficiency were then calculated using the remaining observations.

#### Correction factor approach

The correction factor (CF) approach (23) defined 4 categories of inflammation: *1*) reference (CRP concentration ≤ 5 mg/L and AGP concentration ≤ 1 g/L); *2*) incubation (CRP concentration > 5 mg/L and AGP concentration ≤ 1 g/L); *3*) early convalescence (CRP concentration > 5 mg/L and AGP concentration > 1 g/L); and *4*) late convalescence (CRP concentration ≤ 5 mg/L and AGP concentration > 1 g/L). Survey-specific (i.e., internal) correction factors (ICFs) were calculated by dividing the geometric mean PZC of the reference category by the geometric mean PZC of each inflammatory category, in each survey. The PZC was then multiplied by the appropriate ICF, creating an ICF-adjusted PZC. The prevalence of zinc deficiency in each survey was re-estimated by applying the aforementioned cutoffs to the ICF-adjusted PZC and PROC SURVEY FREQ command. These calculations were performed for CRP alone, AGP alone, and both CRP and AGP.

#### Regression correction approach

The regression correction (RC) approach used linear regression to adjust PZC by CRP, AGP, or both concentrations on a continuous scale, as previously described (25). Linear models were used to maintain consistency with the BRINDA approach, which used regression diagnostics to assess the appropriateness of the linearity assumption between micronutrient biomarkers and CRP or AGP. There was also a desire to employ a modeling approach that could be easily adopted and utilized by others. To summarize, the RC-adjusted PZC was calculated using the following equation:

(1) \begin{equation*} {\rm{PZ}}{{\rm{C}}_{{\rm{RCadjusted}}}} &=& {\rm{PZ}}{{\rm{C}}_{{\rm{unadjusted}}}} - {\beta _1}\left( {\ln \_{\rm{CR}}{{\rm{P}}_{{\rm{obs}}}} - \ln \_{\rm{CR}}{{\rm{P}}_{{\rm{ref}}}}} \right) \\ &&-\, {\beta _2}\left( {\ln \_{\rm{AG}}{{\rm{P}}_{{\rm{obs}}}} - \ln \_{\rm{AG}}{{\rm{P}}_{{\rm{ref}}}}} \right) \end{equation*}

where *β*_1_ is the CRP regression coefficient, *β*_2_ is the AGP regression coefficient, ln_CRP_obs_ is the natural log of the observed CRP value, ln_AGP_obs_ is the natural log of the observed AGP value, ln_CRP_ref_ is the external reference value for the natural log of CRP (PSC: 0.10 mg/L; WRA: 0.16 mg/L), and ln_AGP_ref_ is the external reference value for the natural log of AGP (PSC: 0.59 g/L; WRA: 0.53 g/L). The external reference values were calculated from combined BRINDA project data sets where CRP and AGP assays were sensitive enough to detect concentrations <0.1 mg/L and <0.5 g/L, respectively (25). The RC equation was not applied to individuals that had CRP or AGP concentrations less than or equal to the external reference concentrations because they were considered to have low inflammation. Ecuador was an exception where the survey-specific reference value for CRP was used because the lowest recorded CRP concentration was 1.9 mg/L and constituted >50% of PSC CRP concentrations.

To apply the RC approach, we first replaced any zero CRP or AGP values with values of 0.001 before applying natural-log transformations. In contrast to previous BRINDA analyses of other micronutrient biomarkers (25), we did not log-transform PZC, because it was relatively normally distributed in its original form. Second, to obtain the survey-specific β-coefficients, we used the PROC SURVEYREG command to run linear regression models with PZC as the outcome and ln_CRP_obs_ and/or ln_AGP_obs_ as independent variables. Third, we subtracted the ln_CRP reference value from the observed ln_CRP values, and the ln_AGP reference value from the observed ln_AGP values. Fourth, we multiplied each difference by the corresponding survey-specific β-coefficients, unless it was negative. Finally, we subtracted these 2 products from the unadjusted PZC to obtain the RC-adjusted PZC. Because not all countries measured both CRP and AGP, these calculations were performed using CRP alone, AGP alone, and both CRP and AGP. Once the RC-adjusted PZC was calculated, the prevalence of zinc deficiency was re-estimated by applying the aforementioned cutoffs to the RC-adjusted PZC. Additional information on the RC approach, including a statistical macro for applying this method, can be found at: https://brinda-nutrition.org/the-brinda-approach/.

Prevalence estimates of zinc deficiency were compared among adjustment methods with univariate mixed-model repeated-measures logistic regression (PROC GLIMMIX): the adjustment method was included as a fixed effect; stratum, cluster, and individual subjects were included as random effects; and the observations were weighted according to the survey weights. We did not compare the prevalence of zinc deficiency under the exclusion approach with the other prevalence estimates, because the population of children (i.e., the denominator) was distinctly smaller. Furthermore, we were unable to evaluate the potential role of fasting status, time of blood draw, and other preanalytical factors owing to limited data being available on these factors.

## Results

### Participant characteristics

Our study sample was restricted to participants with no missing values for PZC, and with data available for CRP, AGP, or both. The application of these criteria resulted in a total loss of 4.7% (958 of the 20,236) of the observations that met the BRINDA inclusion criteria in PSC and 1.8% (422 of the 23,383) of the total observations that met the BRINDA inclusion criteria in WRA. An additional 314 PSC observations and 211 WRA observations were excluded on the basis of a PZC above the NHANES maximum (**Supplemental Table 2**), which resulted in final sample sizes of 18,964 PSC and 22,750 WRA.

In PSC, mean age ranged from 20.1 mo in Mongolia to 41.4 mo in Mexico (**Table 1**). The mean age of WRA ranged from 27.2 y in Cameroon to 33.3 y in the United Kingdom. The prevalence of inflammation varied greatly across surveys in PSC (elevated CRP: 8.2–37.5%; elevated AGP: 23.6–55.8%) and in WRA (elevated CRP: 5.2–23.8%; elevated AGP: 7.0–33.5%). The prevalence of inflammation was generally higher in PSC than in WRA. The only exception was Afghanistan, where a greater proportion of WRA than PSC exhibited elevated CRP concentrations. Data on malaria infection were only reported in 2 data sets: in Cameroon, the prevalence of malaria among PSC and WRA was 23.7% and 25.6%, respectively; in Malawi, the prevalence estimates were 27.7% and 17.4%, respectively.

**TABLE 1:** Age and inflammation of PSC and WRA, Biomarkers Reflecting Inflammation and Nutritional Determinants of Anemia project^1^

	Age^2^		CRP			AGP		
Survey, year	*n*	Mean (95% CI)	*n*	Mean (95% CI)	>5 mg/L, % (95% CI)	*n*	Mean (95% CI)	>1 g/L, % (95% CI)
PSC								
Afghanistan, 2013	658	29.0 (27.7, 30.4)	658	1.9 (1.4, 2.3)	9.7 (6.3, 13.1)	658	0.85 (0.81, 0.88)	23.6 (19.3, 28.0)
Azerbaijan, 2013	1016	36.0 (34.8, 37.2)	1016	1.9 (1.4, 2.4)	8.2 (6.1, 10.3)	1016	0.90 (0.87, 0.93)	30.3 (26.3, 34.3)
Bangladesh, 2012	309	36.0 (33.9, 38.1)	309	3.3 (1.1, 5.6)	9.2 (4.2, 14.3)	309	0.87 (0.81, 0.92)	27.8 (20.9, 34.8)
Cambodia, 2014	534	37.4 (36.1, 38.8)	534	2.4 (1.5, 3.3)	11.0 (7.7, 14.2)	534	1.20 (1.05, 1.35)	39.7 (33.4, 46.0)
Cameroon, 2009	776	30.8 (29.8, 31.7)	776	5.4 (4.8, 6.0)	37.5 (32.7, 42.3)	776	0.96 (0.93, 0.98)	38.7 (33.1, 44.3)
Colombia, 2010	3573	37.9 (37.3, 38.5)	3573	4.0 (3.5, 4.5)	18.8 (17.0, 20.6)	—	—	—
Ecuador, 2012	2017	29.3 (28.0, 30.6)	2017	4.0 (3.5, 4.5)	12.5 (10.1, 14.9)	—	—	—
Malawi, 2016	1071	32.3 (30.6, 34.1)	1071	6.3 (5.0, 7.6)	23.8 (19.5, 28.1)	1071	1.35 (1.26, 1.45)	55.8 (50.5, 61.1)
Mexico, 2006	1164	41.4 (40.4, 42.3)	1164	2.1 (1.7, 2.5)	9.8 (7.4, 12.2)	—	—	—
Mongolia, 2006^3^	240	20.1 (19.0, 21.2)	—	—	—	240	0.85 (0.81, 0.88)	24.6
Pakistan, 2011	7231	27.3 (26.9, 27.8)	—	—	—	7231	0.95 (0.94, 0.97)	35.0 (33.6, 36.4)
Vietnam, 2010	375	37.2 (35.9, 38.5)	375	2.6 (1.9, 3.3)	12.3 (9.3, 15.3)	—	—	—
WRA								
Afghanistan, 2013	1044	30.8 (30.2, 31.4)	1044	2.7 (2.2, 3.3)	12.8 (10.4, 15.1)	1043	0.75 (0.72, 0.77)	11.6 (8.7, 14.5)
Bangladesh, 2012	728	29.8 (28.5, 31.1)	727	1.5 (1.2, 1.8)	5.2 (2.8, 7.6)	728	0.74 (0.72, 0.75)	13.1 (8.8, 17.4)
Cambodia, 2014	693	30.2 (29.4, 30.9)	693	1.9 (1.6, 2.3)	9.5 (7.1, 11.9)	693	1.00 (0.87, 1.13)	33.5 (25.2, 41.9)
Cameroon, 2009	746	27.2 (26.5, 27.9)	746	2.8 (2.5, 3.1)	17.8 (14.9, 20.7)	746	0.77 (0.75, 0.78)	7.0 (4.9, 9.1)
Ecuador, 2012	7267	30.2 (29.8, 30.6)	7267	4.0 (3.8, 4.3)	17.2 (15.6, 18.8)	—	—	—
Malawi, 2016	760	27.9 (27.2, 28.6)	760	2.4 (1.7, 3.2)	7.7 (5.4, 10.0)	760	0.69 (0.64, 0.73)	11.3 (8.1, 14.3)
Mexico, 2006	1679	30.5 (29.8, 31.3)	1679	4.0 (3.4, 4.6)	23.8 (20.5, 27.1)	—	—	—
Pakistan, 2011	7490	30.9 (30.8, 31.1)	5616	2.7 (2.4, 2.9)	11.4 (10.4, 12.4)	7488	0.84 (0.83, 0.85)	23.1 (21.8, 24.4)
United Kingdom, 2014	862	33.3 (32.4, 34.2)	862	3.5 (3.0, 4.1)	15.8 (12.4, 19.1)	—	—	—
Vietnam, 2010	1479	32.3 (31.7, 32.8)	1479	1.8 (1.6, 2.0)	6.6 (5.5, 7.8)	—	—	—

1 AGP, α-1-acid glycoprotein; CRP, C-reactive protein; PSC, preschool children; WRA, nonpregnant women of reproductive age.

2 Age values are shown in months for PSC and years for WRA.

3 Not a complex survey design. Values reflect mean (95% CI) or exact proportions.

### Relation between PZC and inflammation

The correlations between the 2 markers of the acute-phase response, CRP and AGP, ranged from 0.58 to 0.75 in PSC and from 0.16 to 0.55 in WRA and were significant (*P* < 0.001) in all surveys (**Table 2**). Overall, PZC tended to be weakly but negatively associated with CRP and AGP concentrations in PSC. In 5 of the 10 PSC data sets that measured PZC and CRP, the Spearman rank correlation coefficient was significant (*P* < 0.05) and ranged from −0.12 to −0.23. The correlation between PZC and AGP was significant (*P* < 0.05) in 5 of the 8 PSC data sets that measured PZC and AGP. In 4 of these 5 surveys, the correlation coefficient ranged from −0.11 to −0.28; however, the association between PZC and AGP was unexpectedly positive in Pakistan (*r* = 0.21, *P* < 0.0001). Associations between PZC and the 2 acute-phase proteins were weak and inconsistent in WRA. The association between PZC and CRP was significant (*P* < 0.05) in only 4 of the 10 WRA surveys. In 3 of these 4 surveys, the correlation coefficient was weak, but negative (*r* = −0.11 to −0.19); however, in Pakistan, it was positive (*r* = 0.04, *P* = 0.02). The association between PZC and AGP in WRA was significant (*P* < 0.05) in 4 of the 6 surveys that measured these biomarkers. However, the correlation was negative in 2 surveys (−0.12, −0.16) and positive in Pakistan (*r* = 0.19, *P* < 0.0001) and Afghanistan (*r* = 0.12, *P* = 0.003).

**TABLE 2:** Weighted Spearman rank correlation coefficients between PZC, CRP, and AGP concentrations in PSC and WRA: Biomarkers Reflecting Inflammation and Nutritional Determinants of Anemia project^1^

	CRP*AGP			PZC*CRP			PZC*AGP		
Survey, year	*n*	*r*	*P*	*n*	*r*	*P*	*n*	*r*	*P*
PSC									
Afghanistan, 2013	658	0.61	<0.0001	658	−0.21	<0.0001	658	−0.15	0.0002
Azerbaijan, 2013	1016	0.67	<0.0001	1016	−0.05	0.08	1016	−0.11	0.005
Bangladesh, 2012	309	0.75	<0.0001	309	−0.05	0.57	309	0.01	0.90
Cambodia, 2014	534	0.58	<0.0001	534	−0.17	0.002	534	−0.15	0.02
Cameroon, 2009	776	0.70	<0.0001	776	−0.23	<0.0001	776	−0.28	<0.0001
Colombia, 2010	—	—	—	3573	0.02	0.29	—	—	—
Ecuador, 2012	—	—	—	2017	−0.14	0.0003		—	—
Malawi, 2016	1071	0.61	<0.0001	1071	−0.12	0.01	1071	−0.03	0.54
Mexico, 2006	—	—	—	1164	−0.004	0.93	—	—	—
Mongolia, 2006		—	—		—	—	240	−0.09	0.16
Pakistan, 2011	—	—	—		—	—	7231	0.21	<0.0001
Vietnam, 2010	—	—	—	375	−0.10	0.05	—	—	—
WRA									
Afghanistan, 2013	1043	0.40	<0.0001	1044	−0.02	0.63	1043	0.12	0.003
Bangladesh, 2012	727	0.50	<0.0001	727	−0.005	0.92	728	0.06	0.54
Cambodia, 2014	693	0.37	<0.0001	693	−0.03	0.44	693	−0.12	0.04
Cameroon, 2009	746	0.55	<0.0001	746	−0.19	<0.0001	746	−0.16	<0.0001
Ecuador, 2012	—	—	—	7267	−0.11	<0.0001	—	—	—
Malawi, 2016	760	0.47	<0.0001	760	−0.03	0.62	760	−0.02	0.65
Mexico, 2006	—	—	—	1679	0.03	0.53	—	—	—
Pakistan, 2011	5614	0.16	<0.0001	5616	0.04	0.02	7488	0.19	<0.0001
United Kingdom, 2014	—	—	—	862	−0.16	0.001	—	—	—
Vietnam, 2010	—	—	—	1479	−0.002	0.93	—	—	—

1 *P* values were calculated from a regression model that accounted for complex sampling (cluster, strata, and sampling weight). AGP, α-1-acid glycoprotein; CRP, C-reactive protein; PSC, preschool children; PZC, plasma or serum zinc concentration; WRA, nonpregnant women of reproductive age.

**FIGURE 1:**
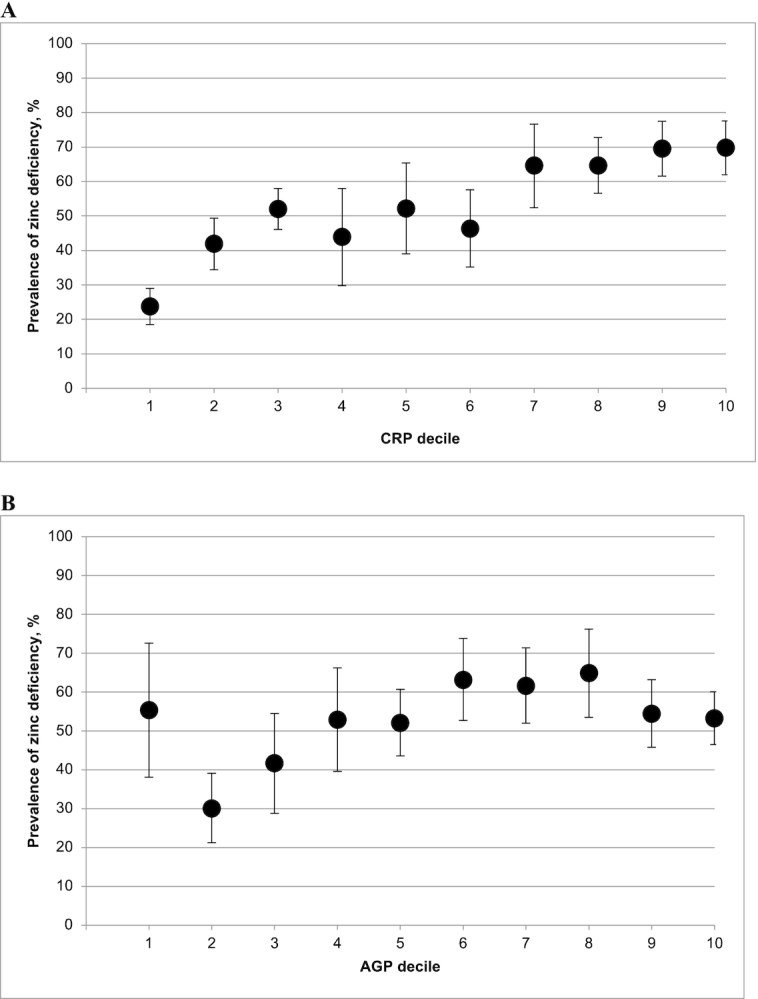
Estimated prevalence [% (95% CI)] of zinc deficiency in PSC by CRP (A) and AGP (B) deciles. The analysis was restricted to countries that measured both CRP and AGP: Afghanistan, Azerbaijan, Bangladesh, Cambodia, Cameroon, and Malawi, for comparability between CRP and AGP relations with zinc deficiency. Zinc deficiency is defined as a plasma or serum zinc concentration <57 *µ*g/dL in the afternoon or <65 *µ*g/dL in the morning (nonfasting) in PSC. AGP, α-1-acid glycoprotein; CRP, C-reactive protein; PSC, preschool children.

**FIGURE 2:**
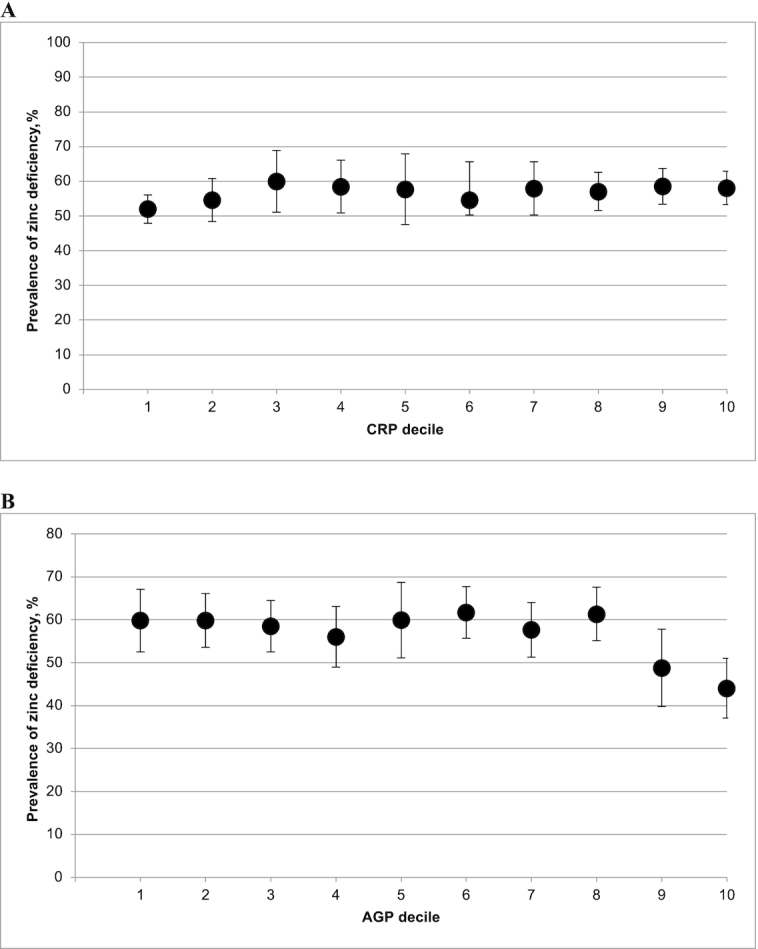
Estimated prevalence [% (95% CI)] of zinc deficiency in WRA by CRP (A) and AGP (B) deciles. The analysis was restricted to countries that measured both CRP and CRP: Afghanistan, Bangladesh, Cambodia, Cameroon, and Malawi. Zinc deficiency is defined as a plasma or serum zinc concentration <59 *µ*g/dL in the afternoon, <66 *µ*g/dL in the morning (nonfasting), or <70 *µ*g/dL in the morning (fasting) in WRA. AGP, α-1-acid glycoprotein; CRP, C-reactive protein; WRA, women of reproductive age.

The relation between the estimated prevalence of zinc deficiency and CRP decile visually appeared to follow a positive linear pattern in the pooled analysis of the 6 PSC data sets that measured both CRP and AGP concentrations (**Figure 1**A). Although the association was less clear, the estimated prevalence of zinc deficiency also tended to be higher as AGP decile increased (Figure 1B). However, country-specific analyses revealed considerable variation in the nature of the association between the prevalence of zinc deficiency and the acute-phase protein decile in PSC (**Supplemental Figure 2**A). No clear pattern could be identified between the prevalence of zinc deficiency and either CRP decile or AGP decile in the pooled analysis of the 5 WRA data sets that measured both CRP and AGP concentrations (**Figure 2**), and the same was true for most of the country-specific analyses (Supplemental Figure 2B).

Based on the results of the correlation and decile analyses, we determined that it was appropriate to pursue and compare adjustment methods for PZC in the following PSC data sets: Cameroon, Malawi, Ecuador, Afghanistan, Cambodia, and Azerbaijan. We determined that adjustment methods were not warranted in the case of WRA given the lack of a strong or consistent association between PZC and either CRP or AGP.

### Inflammation CFs for PZC in PSC

Among the 5 PSC surveys that measured both CRP and AGP and were selected for adjustment, the geometric mean PZC of 62.5 *µ*g/dL was highest in PSC with no inflammation (**Table 3**). The geometric mean was 53.4 *µ*g/dL among children in the incubation period, 50.7 *µ*g/dL among children in early convalescence, and 59.2 *µ*g/dL during late convalescence, all of which were significantly lower than the reference category. With the exception of Malawi, ICFs for each stage of the acute-phase response ranged between 1.02 and 1.24 in all 5 surveys that were analyzed (**Supplemental Table 3**A). The values of the ICFs were similar when calculated based on elevated CRP concentration or elevated AGP concentration alone (Supplemental Table 3B).

**TABLE 3:** PZCs in preschool children according to inflammation stage^1^

Inflammation stage	*n*	PZC, μg/dL
Reference (CRP ≤ 5 mg/L and AGP ≤ 1 g/L)	2356	62.5 (61.1, 64.0)
Incubation (CRP > 5 mg/L and AGP ≤ 1 g/L)	124	53.4 (51.3, 55.6)
Early convalescence (CRP > 5 mg/L and AGP > 1 g/L)	646	50.7 (49.1, 52.4)
Late convalescence (CRP ≤ 5 mg/L and AGP > 1 g/L)	929	59.2 (57.4, 61.1)

1 Values are geometric mean (95% CI) unless otherwise indicated. Only includes data from Afghanistan, Azerbaijan, Cambodia, Cameroon, and Malawi. AGP, α-1-acid glycoprotein; CRP, C-reactive protein; PZC, plasma or serum zinc concentration.

### RC slopes for PZC and inflammation in PSC

Bivariate linear regression with PZC as the outcome resulted in an unstandardized ln-CRP slope that ranged from −0.36 (*P* = 0.15) in Azerbaijan to −2.75 (*P* = 0.004) in Ecuador, and an unstandardized ln-AGP slope that ranged from −0.93 (*P* = 0.57) in Malawi to −13.780 (*P* < 0.001) in Cameroon (**Supplemental Table 4**A). A multivariate analysis of PZC with both ln-CRP and ln-AGP in the model tended to attenuate the magnitude of the ln-CRP slope and ln-AGP slope.

### Estimated prevalence of zinc deficiency by inflammation adjustment method

**FIGURE 3:**
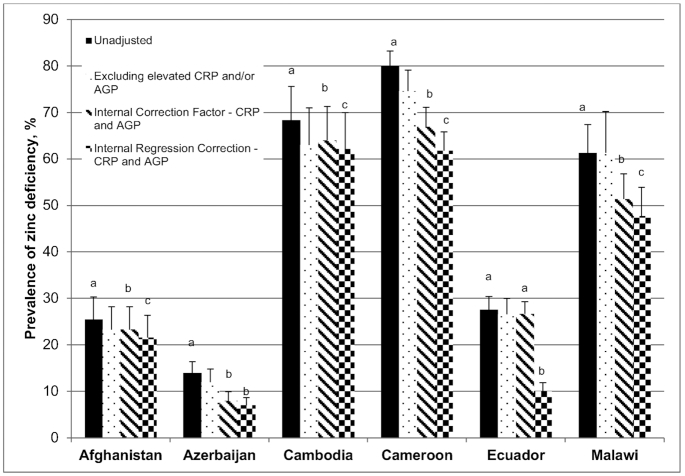
Estimated prevalence [% (95% CI)] of zinc deficiency with the use of different adjustment approaches in PSC. Zinc deficiency is defined as a plasma or serum zinc concentration <57 *µ*g/dL in the afternoon or <65 *µ*g/dL in the morning (nonfasting) in PSC. Bars within a given survey without a common letter are significantly different, *P* < 0.05 (adjusted for multiple comparisons). Statistical comparisons were not made with the prevalence of zinc deficiency under the exclusion approach, because the population of PSC is different. Adjustment methods only account for CRP in Ecuador. AGP, α-1-acid glycoprotein; CRP, C-reactive protein; PSC, preschool children.

There was drastic variation in the prevalence of unadjusted zinc deficiency across the 6 PSC surveys that were pursued for adjustment (**Figure 3**). The estimated prevalence ranged from a low of 14.0% in Azerbaijan to a high of 80.0% in Cameroon. Not surprisingly, the prevalence of zinc deficiency tended to be higher in countries that also demonstrated a high prevalence of inflammation.

The exclusion of PSC with elevated CRP or AGP reduced the sample size of each survey by 8–59% (**Supplemental Table 5**). The exclusion of observations with CRP concentrations > 5 mg/L resulted in a small decrease in the estimated prevalence of zinc deficiency, of 0.7–3.9 percentage points (pp). The magnitude of the reduction was comparable when exclusions were based only on AGP concentrations > 1 g/L. Excluding observations with elevated CRP or AGP concentrations resulted in the largest reduction in sample size; however, the change in the prevalence of zinc deficiency remained small. The greatest reduction in the estimated prevalence was observed in Cameroon, where the prevalence of zinc deficiency fell by 5.4 pp.

The estimated prevalence of zinc deficiency decreased when ICFs for CRP only, AGP only, and both CRP and AGP were used to adjust the PZC across all 6 surveys (Supplemental Table 3C). In most cases, the greatest reduction was observed when ICFs for both CRP and AGP were applied. The magnitude of the reduction ranged from 2.2 pp in Afghanistan to 13.1 pp in Cameroon.

Similarly, the application of the BRINDA RC approach reduced the estimated prevalence of zinc deficiency in all 6 PSC surveys that were analyzed (Supplemental Table 4B). In Cameroon, Malawi, and Cambodia, the bivariate model including CRP only and the multivariate model including both CRP and AGP produced similar adjusted prevalence estimates of zinc deficiency; however, the bivariate model including AGP only produced a smaller decrease in the estimated prevalence of zinc deficiency. In Afghanistan and Azerbaijan, there was very little difference in the 3 adjusted prevalence estimates. Overall, the application of any of the RC models including both CRP and AGP reduced the estimated prevalence of zinc deficiency by 4.1–18.2 pp.

### Comparison of the estimated prevalence of zinc deficiency in PSC with the use of different approaches to adjust PZC for inflammation

Comparisons across adjustment approaches were made using the variations that accounted for both CRP and AGP for the following reasons: *1*) with the exception of Ecuador, which only had data available for CRP, both variables were available in the surveys that were analyzed; *2*) the greatest reduction in prevalence of zinc deficiency was usually seen when both variables were accounted for; *3*) each variable represents a different phase of the acute-phase response and is important from a biological perspective. In Cameroon and Malawi, the CF approach using both CRP and AGP significantly (*P* < 0.05) reduced the estimated prevalence of deficiency by 13.1 and 9.6 pp, respectively (Figure 3). With the exception of Ecuador, in all other countries the magnitude of the reduction was significant, but considerably smaller. In all 6 surveys, application of the RC approach, adjusting for both CRP and AGP when available, resulted in the largest reduction from the unadjusted prevalence of zinc deficiency; this reduction was consistently significant. In Cameroon, the estimated prevalence of zinc deficiency fell by >15 pp. In Malawi, Cambodia, Ecuador, and Azerbaijan, the estimated prevalence dropped by 5–15 pp, and in Afghanistan, the change was <5 pp.

## Discussion

The results of this large, multicountry analysis showed that inflammation was associated with reduced PZC and appeared to produce artificially high prevalence estimates of nutritional zinc deficiency in a subset of PSC surveys. The prevalence of zinc deficiency in surveys where inflammation adjustments were pursued decreased by a median of 7.1 pp. The association between inflammation and PZC in WRA was weak and inconsistent. To our knowledge, our analysis is the first to systematically evaluate different methods of adjusting PZC for the effects of inflammation, using nationally representative survey data collected from PSC in 12 countries with a broad range of both zinc deficiency and inflammation.

The first objective of this analysis was to determine whether it is necessary to adjust PZC for inflammation to estimate the prevalence of nutritional zinc deficiency among PSC and WRA. Our pooled decile analysis of PSC data sets that measured both CRP and AGP revealed a clear positive linear association between the prevalence of zinc deficiency and CRP decile, and a generally higher prevalence of zinc deficiency among higher AGP deciles. However, those findings masked sizeable differences in the relation between PZC and inflammation between surveys. Survey-specific analyses revealed a significant correlation coefficient <−0.10 between PZC and CRP or AGP in PSC from Cameroon, Malawi, Afghanistan, Cambodia, Azerbaijan, and Ecuador. However, in Bangladesh, Colombia, Mexico, Vietnam, and Mongolia, the correlation was weak and/or nonsignificant, and in Pakistan, the association between PZC and AGP was weakly positive. Survey-specific analyses of the prevalence of zinc deficiency according to CRP or AGP decile were generally consistent with the results of the correlation analyses. Given such variation across surveys, both correlation and decile analyses should be conducted with data from a particular population of PSC. The decision to apply adjustment methods should consider the strength and significance of the correlation coefficient between PZC and CRP or AGP, and should also include a visual inspection of the decile analysis to confirm an increasing prevalence of zinc deficiency by CRP or AGP decile. Our pooled and survey-specific analysis of WRA data sets did not reveal a clear relation between PZC and CRP or AGP. Based on this currently available evidence, we determined that methods to adjust PZC for inflammation among WRA are not warranted at this time. However, it may be helpful to conduct the correlation and decile analyses as part of the initial data analysis process when analyzing data from WRA.

Our second objective was to determine whether it is necessary to adjust PZC for CRP, AGP, or both in populations where adjustment is warranted. CRP concentrations rise quickly and acutely in response to infection, whereas elevated AGP concentrations are indicative of longer-term exposure to inflammation (14). Adjustment methods that only used CRP often reduced the prevalence of zinc deficiency in PSC by a similar degree to methods that accounted for both CRP and AGP. However, because AGP represents an important part of the acute-phase response, and understanding the relation between PZC and the acute-phase proteins is an emerging area, it is likely useful to measure and include AGP with CRP in the adjustment method for PSC while research in this area continues.

Our third objective was to compare the ability of adjustment approaches to estimate PZC in the presence of inflammation and estimate the prevalence of nutritional zinc deficiency. We pursued this objective in the subset of 6 PSC data sets that demonstrated a significant negative association of PZC with CRP and/or AGP. As with the previous BRINDA articles (16, 18–20), we considered the following 3 criteria to compare the adjustment approaches: *1*) precision; *2*) variability reflecting the relation between nutrient biomarkers and severity of inflammation; and *3*) feasibility to implement across countries. There was substantial variability in the degree to which the different adjustment approaches affected the estimated prevalence of zinc deficiency. Although the exclusion approach is the simplest adjustment method, its application leads to large reductions in sample size and corresponding precision. It is also possible that the exclusion of such a large proportion of subjects may introduce bias (26). Although the ICF approach accounts for the inflammation profile of the population being analyzed, the precision of the adjusted prevalence estimate of zinc deficiency is dependent on the size of the reference group. Because the RC approach applies a more stringent adjustment technique, its application resulted in the largest decline in the estimated prevalence of zinc deficiency. The RC approach has the advantage of adjusting for CRP and AGP concentrations on a linear scale, and by using survey-specific slopes, we were able to account for considerable variation in the strength of the association of PZC with CRP and/or AGP. However, the external CRP and AGP reference deciles that we applied may not be suitable in all contexts. Furthermore, given the lack of a gold-standard biomarker of zinc status, we cannot be certain that this approach provides an accurate estimate of the prevalence of nutritional zinc deficiency that would exist in the absence of inflammation.

The fact that a negative association of PZC with CRP and AGP did not occur in all of the PSC data sets is puzzling. Reductions in PZC have been observed in animal and human studies involving the experimental induction of infection (27, 28). Under such conditions, hepatic metallothionein is induced, which leads to a redistribution of plasma zinc to the liver (12, 27–30). Although some studies have shown a relation between PZC and markers of inflammation in PSC (12, 31), a review of 3 cross-sectional community-based studies involving PSC in Peru, Guatemala, and Zimbabwe did not find any significant differences in mean PZC between infected and noninfected children (10). Some evidence from clinical studies indicates that the magnitude of the change in PZC may be related to the severity and stage of infection (32, 33), which could explain some of the variation in the association between PZC and inflammation across data sets included in our analysis. Also, in national surveys, it is common practice to include only apparently healthy subjects, which thereby excludes more severe infections. It is worth noting that the adjustment methods had the greatest impact on the prevalence of zinc deficiency among PSC in Cameroon, the country that demonstrated the highest unadjusted prevalence of zinc deficiency, highest mean CRP concentration, and among the highest mean AGP concentrations. However, the unadjusted prevalence of zinc deficiency and the prevalence of elevated AGP were also very high in Pakistani PSC, yet the opposite association between AGP and PZC was observed. It is also possible that the severity of infection was lower in WRA, as evidenced by generally lower CRP and AGP concentrations, which could explain the weak and inconsistent effect of inflammation on PZC in WRA. Without having more information on potential effect modifiers, such as the type and severity of infection and confirmed pregnancy status, it is difficult to determine possible reasons for the discrepant findings between countries. Research by Duggan et al. has shown that malaria parasite density is independently and negatively associated with PZC (11); however, Wessells et al. did not find any significant differences in PZC between children with and without elevated histidine-rich protein 2 of the malaria parasite *Plasmodium falciparum* (34). Unfortunately, data on malaria infection were only reported in 2 of the 13 surveys that were included in our analysis, so we were not able to systematically investigate the effect of this potentially important variable.

One of the key limitations of our analysis is the variability in the conditions and methods that were used to obtain blood samples and analyze PZC, CRP, and AGP concentrations. It is concerning that 4.4% and 7.2% of the PZC observations from PSC in Colombia and Mexico, respectively, were above the maximum PZC observed in NHANES; however, contamination was not confirmed. Although we excluded these observations from analysis assuming contamination, this does not address the possible sources of contamination that could have systematically biased all PZC values in a particular survey upwards. Because this is a secondary analysis of existing data sets, we are also limited by the micronutrient and inflammation biomarker data that are available. Our analysis was particularly constrained by a lack of information about CRP in the data sets, for which the limit of detection (LOD)/limit of quantification (LOQ) can vary widely across assays and for which a high proportion of undetectable or unquantifiable observations may be observed. In a sensitivity analysis involving multiple surveys that used a single laboratory for analysis of CRP, we replaced CRP values below the known LOD of 0.5 mg/L with a value of 0.25 mg/L and reran the correlation analysis. As shown in **Supplemental Table 6**, the results of this sensitivity analysis yielded similar results to the primary analysis. Unfortunately, information on the LOD/LOQ was not available for all surveys and future work should investigate how different approaches for handling values below the LOD/LOQ may influence the interpretation of biomarkers.

It is possible that some of the blood samples may have been hemolyzed, which would have caused an increase in PZC values (35). We are also limited by the fact that PZC is an imperfect measure of zinc status and can be influenced by many factors, such as the time of day and time of the last meal consumption, which were not precisely recorded in all surveys. It is important to keep in mind the limitations of the biomarkers that were included in our analysis and the fact that measurable changes in PZC, CRP, and AGP may not occur in the presence of subclinical inflammation, even with low dietary intake of zinc (36). We encourage further investigation of alternative biomarkers of inflammation, such as IL-6, which has been shown to be a more sensitive indicator of chronic inflammation and may be suitable for use in adjusting micronutrient status in some populations (30). It is also not possible to rule out the possibility of reverse causation that is present in cross-sectional studies, and the fact that children with higher zinc status could be less prone to infection and inflammation. These limitations call for further analyses involving longitudinal studies with high-quality biomarker data and comprehensive assessment of variables that could potentially confound or modify the association between PZC and inflammation.

In conclusion, our study provides important insights into the relation between PZC and inflammation in PSC and WRA. Our findings emphasize the importance of performing correlation and decile analyses to understand the strength and direction of the association, and we encourage the use of the RC method to adjust PZC for both CRP and AGP concentrations in PSC populations where adjustment is necessary. Based on our results, adjusting PZC for inflammation in WRA does not appear necessary; however, correlation and decile analyses can still be a useful part of data processing and analysis in this population. The results of our analyses are relevant from a public health perspective, because the estimated prevalence of deficiency guides decision-making on the need for, and the design, targeting, and evaluation of, interventions to improve zinc nutritional status.

## Supplementary Material

nqz304_Supplemental_File

## Notes

Supported by the Bill & Melinda Gates Foundation grant OPP1158306, Centers for Disease Control and Prevention, *Eunice Kennedy Shriver* National Institute of Child Health and Human Development, HarvestPlus, and the United States Agency for International Development.

Supplemental Tables 1–6 and Supplemental Figures 1 and 2 are available from the “Supplementary data” link in the online posting of the article and from the same link in the online table of contents at https://academic.oup.com/ajcn/.

Data described in the article, code book, and analytic code will be made available upon request pending approval from the BRINDA steering committee and country representatives.

Published in a supplement to *The American Journal of Clinical Nutrition*. Publication costs for this supplement were defrayed in part by the payment of page charges and supported by the Bill & Melinda Gates Foundation. The Guest Editor for this supplement was Daniel E Roth and has no relevant conflicts to disclose. The opinions expressed in this publication are those of the authors and are not attributable to the sponsors or the publisher, Editor, or Editorial Board of *The American Journal of Clinical Nutrition*. The Supplement Coordinators for the supplement publication were Parminder S Suchdev and Melissa F Young, Emory University, Atlanta, GA 30322. Supplement Coordinator disclosures: PSS receives salary support from the CDC; MFY, no conflicts to disclose.

Abbreviations used: AGP, α-1-acid glycoprotein; BRINDA, Biomarkers Reflecting Inflammation and Nutritional Determinants of Anemia; CF, correction factor; CRP, C-reactive protein; ICF, internal correction factor; IZiNCG, International Zinc Nutrition Consultative Group; LOD, limit of detection; LOQ, limit of quantification; pp, percentage points; PSC, preschool children; PZC, plasma or serum zinc concentration; RC, regression correction; WRA, nonpregnant women of reproductive age 15–49 y.
