# Supplementary material for: Adjusting plasma or serum zinc concentrations for inflammation: Biomarkers Reflecting Inflammation and Nutritional Determinants of Anemia (BRINDA) project
Source: Am J Clin Nutr. 2020 Apr 8;111(4):927–37. doi: 10.1093/ajcn/nqz304 (PMC7138668; doi:10.1093/ajcn/nqz304)
Supplement: nqz304_Supplemental_File [file nqz304_supplemental_file.zip › BRINDA zinc& inflammation AJCN OSM FIGS 10212019.docx]

**Supplementary Figures to *Adjusting plasma or serum zinc concentrations for inflammation: Biomarkers Reflecting Inflammation and Nutritional Determinants of Anemia (BRINDA) project* by McDonald et al.**

**Supplementary Figure 1. BRINDA Project Available Data for Analysis of Zinc and Inflammation**

**BRINDA Phase 1 & 2**

24 PSC datasets (N=85,458);

19 WRA datasets (N= 107,393)

**Data Included in Analysis**

12 PSC datasets (N = 18,964);

10 WRA datasets (N = 22,748)

**Inclusion Criteria**

At least one inflammation biomarkers *AND*

Plasma or Serum Zinc Concentration *AND* Dataset *n* > 100

**Supplementary Figure 2A. Country-specific prevalence of zinc deficiency by CRP and AGP decile in preschool children^1^**

**Cameroon**

**Colombia**

**Mexico**

**Pakistan**

**Malawi**

**Ecuador**

**Afghanistan**

**Bangladesh**

**Cambodia**

**Mongolia**

**Vietnam**

**Azerbaijan**

^1^alpha-1-acid glycoprotein: AGP; C-reactive protein: CRP

**Supplementary Figure 2B. Country-specific prevalence of zinc deficiency by CRP and AGP decile in women of reproductive age**^1^

**Cameroon**

**Mexico**

**Pakistan**

**Malawi**

**Ecuador**

**Afghanistan**

**Bangladesh**

**Cambodia**

**Vietnam**

**United Kingdom**

^1^alpha-1-acid glycoprotein: AGP; C-reactive protein: CRP
