# Supplementary material for: Adjusting plasma or serum zinc concentrations for inflammation: Biomarkers Reflecting Inflammation and Nutritional Determinants of Anemia (BRINDA) project
Source: Am J Clin Nutr. 2020 Apr 8;111(4):927–37. doi: 10.1093/ajcn/nqz304 (PMC7138668; doi:10.1093/ajcn/nqz304)
Supplement: nqz304_Supplemental_File [file nqz304_supplemental_file.zip › BRINDA zinc& inflammation AJCN OSM TBLS 10222019.docx]

**Supplementary Tables to *Adjusting plasma or serum zinc concentrations for inflammation: Biomarkers Reflecting Inflammation and Nutritional Determinants of Anemia (BRINDA) project* by McDonald et al.**

**Supplementary Table 1. Summary of sample collection and laboratory methods^1^**

| **Survey** | **Fasting status** | **Venous or capillary blood** | **Plasma or serum** | **Time of blood draw** | **Time of Separation** | **Zinc analysis laboratory** | **Zinc analysis method** | **CRP analysis method** | **AGP analysis method** |
| --- | --- | --- | --- | --- | --- | --- | --- | --- | --- |
| Afghanistan | Not collected | Venous | Serum | Not collected | Unknown | Aga Khan University, Karachi, Pakistan | AAS | Immunoassay | Turbidimetry |
| Azerbaijan | Not collected | Venous | Plasma | Recorded | Unknown | Children’s Hospital Oakland Research Institute, USA | ICP-OES | ELISA | ELISA |
| Bangladesh | Not collected | Venous | Serum | Not collected | Unknown | icddr,b, Dhaka, Bangladesh | AAS | ELISA | ELISA |
| Cambodia | Not collected | Venous | Plasma | Recorded: AM | PM  (same day) | National Institute of Nutrition, Vietnam | AAS | ELISA | ELISA |
| Cameroon | Mostly fasted | Venous | Plasma | Recorded: 7am-3pm | Within 2 hours of collection | Children’s Hospital Oakland Research Institute, USA | ICP-OES | ELISA | ELISA |
| Colombia | Unknown | Venous | Plasma | Unknown | Unknown | National Institute of Health of Colombia | AAS | Turbidimetry | N/A |
| Ecuador | Fasted for children > 2y | Venous | Serum | Yes | Unknown | Unknown | AAS | Nephelometry | N/A |
| Malawi | Yes | Venous | Serum | Recorded | Unknown | Children’s Hospital Oakland Research Institute, USA | ICP-OES | ELISA | ELISA |
| Mexico | Fasted | Venous | Serum | Not recorded | Not collected | Laboratorio del Centro de Investigación en Nutrición y Salud del INSP | ICP-OES | Nephelometry | N/A |
| Mongolia | Non-fasting | Capillary/  Venous | Serum | Recorded: AM | Unknown | University of Otago, New Zealand | AAS | N/A | Turbidimetry |
| Pakistan | Unknown | Venous | Serum | Unknown | Unknown | Aga Khan University, Karachi, Pakistan | AAS | Immunoassay | Turbidimetry |
| United Kingdom | Fasting | Venous | Unknown | Not recorded | Unknown | MRC, Cambridge, UK | ICP-MS | ELISA | N/A |
| Vietnam | Non-fasting | Venous | Serum | Recorded: AM | PM  (same day) | National Institute of Nutrition, Vietnam | AAS | ELISA | N/A |

^1^alpha-1-acid glycoprotein (AGP); C-reactive protein (CRP); N/A indicates that biomarker was not analyzed in that survey.

**Supplementary Table 2. Proportion of PZC values above the NHANES maximum that were excluded from analysis, by country^1^**

| **Survey** | **PZC observations > NHANES maximum**  **N (%)** |
| --- | --- |
| **Preschool children** |  |
| Afghanistan | 1 (0.01%) |
| Azerbaijan | 3 (0.02%) |
| Bangladesh | 1 (0.01%) |
| Cambodia | 0 (0.0%) |
| Cameroon | 0 (0.0%) |
| Colombia | 185 (4.4%) |
| Ecuador | 1 (0.01%) |
| Malawi | 9 (0.8%) |
| Mexico | 90 (7.2%) |
| Mongolia | 0 (0.0%) |
| Pakistan | 43 (0.6%) |
| Vietnam | 0 (0.0%) |
| **Women of reproductive age** |  |
| Afghanistan | 4 (1.9%) |
| Bangladesh | 0 (0.0%) |
| Cambodia | 0 (0.0%) |
| Cameroon | (0.0%) |
| Ecuador | 1 (0.01%) |
| Malawi | 8 (1.0%) |
| Mexico | 144 (7.9%) |
| Pakistan | 50 (0.7%) |
| Vietnam | 2 (1.0%) |
| United Kingdom | 2 (1.0%) |

^1^Nutrition and Health Examination Survey (NHANES); plasma or serum zinc concentration (PZC)

**Supplementary Table 3a. Survey-specific geometric mean of plasma zinc according to 4 inflammation stages, and internal correction factors in preschool children**

|  | **Healthy** | **Incubation** | | **Early convalescence** | | **Late convalescence** | |
| --- | --- | --- | --- | --- | --- | --- | --- |
| **Survey** | **Geometric mean, μg/dL**  **(95% CI)** | **Geometric mean, μg/dL**  **(95% CI)** | **ICF**^1^ | **Geometric mean, μg/dL**  **(95% CI)** | **ICF**^1^ | **Geometric mean, μg/dL**  **(95% CI)** | **ICF**^1^ |
| Afghanistan | 75.0  (72.2, 77.9) | 69.9  (59.3, 82.4) | 1.07 | 69.3  (63.6, 75.5) | 1.08 | 72.0  (67.9, 76.3) | 1.04 |
| Azerbaijan | 71.2  (69.9, 72.6) | 66.6  (61.4, 72.3) | 1.07 | 66.4  (61.3, 71.8) | 1.07 | 69.8  (67.7, 72.1) | 1.02 |
| Cambodia | 58.4  (55.5, 61.4) | 53.5  (34.3, 83.4) | 1.09 | 47.0  (41.9, 52.7) | 1.24 | 54.4  (50.2, 58.9) | 1.07 |
| Cameroon | 53.5  (52.3, 54.6) | 50.9  (48.5, 53.3) | 1.05 | 47.2  (45.1, 49.3) | 1.13 | 49.5  (47.2, 51.8) | 1.08 |
| Malawi | 57.2  (54.9, 59.6) | 61.2  (43.5, 86.1) | 0.93 | 52.7  (49.8, 55.7) | 1.09 | 58.9  (56.5, 61.4) | 0.97 |

^1^Internal correction factors (ICF) were calculated by dividing the geometric mean of the healthy category by the geometric mean of each inflammation stage.

**Supplementary Table 3b. Geometric mean of plasma zinc in preschool children according to CRP concentration, AGP concentration, and corresponding internal correction factors (ICF)^1^**

| **Survey** | **CRP ≤ 5 mg/L** | **CRP > 5 mg/L** | **ICF**^2^ | **AGP ≤ 1 g/L** | **AGP > 1 g/L** | **ICF**^3^ |
| --- | --- | --- | --- | --- | --- | --- |
|  | **Geometric mean**  **(95% CI)** | **Geometric mean**  **(95% CI)** |  | **Geometric mean**  **(95% CI)** | **Geometric mean**  **(95% CI)** |  |
| Afghanistan | 74.5  (72.0, 77.1) | 69.4  (65.1, 74.0) | 1.07 | 74.9  (72.1, 77.7) | 71.1  (67.9, 74.5) | 1.05 |
|  |  |  |  |  |  |  |
| Azerbaijan | 70.9  (69.7, 72.1) | 66.4  (61.9, 71.2) | 1.07 | 71.2  (69.9, 72.5) | 69.0  (66.6, 71.5) | 1.03 |
| Cambodia | 57.0  (54.1, 59.9) | 48.3  (44.2, 52.7) | 1.18 | 58.2  (55.4, 61.2) | 52.7  (48.7, 56.9) | 1.10 |
| Cameroon | 52.8  (51.7, 53.8) | 48.1  (46.3, 49.9) | 1.10 | 53.1  (52.0, 54.2) | 47.8  (46.1, 49.5) | 1.11 |
| Ecuador | 71.7  (70.8, 72.6) | 67.5  (64.8, 70.3) | 1.06 | --- | --- | --- |
| Malawi | 57.9  (56.1, 59.8) | 53.0  (50.3, 55.9) | 1.09 | 57.3  (54.9, 59.7) | 56.3  (53.9, 58.8) | 1.02 |

^1^alpha-1-acid glycoprotein: (AGP); C-reactive protein (CRP)

^2^Internal correction factor (ICF) was calculated by dividing the geometric mean serum zinc concentration in the low CRP category by the geometric mean serum zinc concentration in the high CRP category

^3^Internal correction factor was calculated by dividing the geometric mean serum zinc concentration in the low AGP category by the geometric mean serum zinc concentration in the high AGP category

**Supplementary Table 3c. Prevalence of zinc deficiency in preschool children after applying internal correction factors (ICF)**^1,2^

| **Survey** | **Unadjusted**  **%**  **(95% CI)** | **ICF:**  **CRP only**  **%**  **(95% CI)** | **ICF:**  **AGP only**  **%**  **(95% CI)** | **ICF:**  **CRP and AGP**  **%**  **(95% CI)** |
| --- | --- | --- | --- | --- |
| Afghanistan | 25.5^a^  (20.6, 30.3) | 23.9^b^  (19.1, 28.6) | 23.6^b^  (18.5, 28.6) | 23.3^b^  (18.4, 28.2) |
| Azerbaijan | 14.0^a^  (11.5, 16.4) | 8.4^b^  (6.4, 10.3) | 8.7^b^  (6.7, 10.7) | 8.0^b^  (6.1, 9.9) |
| Cambodia | 68.3^a^  (61.0, 75.6) | 66.4^a,b^  (59.1 73.7) | 64.1^c^  (56.6, 71.6) | 64.0^c^  (56.7, 71.3) |
| Cameroon | 80.0^a^  (76.7, 83.3) | 71.6 ^b^  (68.0, 75.2) | 69.0^b,c^  (65.2, 72.9) | 66.9^c^  (62.6, 71.1) |
| Ecuador | 27.6^a^  (24.7, 30.4) | 26.7^b^  (24.0, 29.3) | --- | --- |
| Malawi | 61.3^a^  (55.2, 67.4) | 52.6^b,d^  (46.0, 59.2) | 50.8^c,d^  (44.4, 57.2) | 51.7^d^  (46.3, 57.2) |

^1^Zinc deficiency is defined as a PZC <59 µg/dL in afternoon or < 66 µg/dL in morning (non-fasting), <70 µg/dL in morning (fasting) in women of reproductive age. Prevalence estimates in the same row without the same superscript are statistically significant different from one another (p<0.05, adjusted using Bonferroni correction) using PROC GLIMMIX.

^2^alpha-1-acid glycoprotein (AGP); C-reactive protein (CRP); plasma or serum zinc concentration (PZC)

**Supplementary Table 4a. Beta coefficients for logCRP and logAGP in country-specific regression models including preschool children^1^**

|  | **Model including logCRP and logAGP** | | **Model including logCRP only** | **Model including logAGP only** |
| --- | --- | --- | --- | --- |
|  | **β(logCRP)** | **β(logAGP)** | **β(logCRP)** | **β(logAGP)** |
| Afghanistan | -0.981  p=0.003 | -0.549  p=0.77 | -1.011  p=0.0005 | -5.432  p=0.005 |
| Azerbaijan | 0.086  p=0.78 | -4.101  p=0.09 | -0.358  p=0.15 | -3.771  p=0.04 |
| Cambodia | -1.779  p=0.001 | -0.763  p=0.56 | -2.011  p=0.0003 | -2.642  p=0.03 |
| Cameroon | -0.279  p=0.46 | -12.408  p<0.0001 | -1.444  p<0.0001 | -13.795  p<0.0001 |
| Ecuador | --- | --- | -2.75  p=0.0004 | --- |
| Malawi | -1.849  p=0.003 | 2.600  p=0.23 | -1.353  p=0.001 | -0.929  p=0.57 |

^1^alpha-1-acid glycoprotein (AGP); C-reactive protein (CRP)

**Supplementary Table 4b. Prevalence of zinc deficiency in preschool children based on BRINDA internal regression correction**^1,2^

| **Survey** | **Unadjusted**  **%**  **(95% CI)** | **RC:**  **CRP only**  **%**  **(95% CI)** | **RC:**  **AGP only**  **%**  **(95% CI)** | **RC:**  **CRP and AGP**  **%**  **(95% CI)** |
| --- | --- | --- | --- | --- |
| Afghanistan | 25.5^a^  (20.6, 30.3) | 22.0^b^  (17.3, 26.6) | 21.4^b^  (17.0, 25.8) | 21.4^b^  (17.0, 25.8) |
| Azerbaijan | 14.0^a^  (11.5, 16.4) | 8.4^b^  (6.4, 10.3) | 7.0^b^  (5.2, 8.7) | 7.0^b^  (5.2, 8.7) |
| Cambodia | 68.3^a^  (61.0, 75.6) | 62.3^b,d^  (54.4, 70.2) | 66.0^c^  (58.2, 73.8) | 62.1^d^  (54.2, 70.0) |
| Cameroon | 80.0^a^  (76.7, 83.3) | 63.2^b,d^  (59.1, 67.2) | 70.1^c^  (66.2, 74.1) | 61.8^d^  (57.8, 65.8) |
| Ecuador | 27.6^a^  (24.7, 30.4) | 10.0^b^  (8.1, 11.9) | --- | --- |
| Malawi | 61.3^a^  (55.2, 67.4) | 45.6^b,d^  (39.5, 51.7) | 51.0^c^  (44.6, 57.3) | 47.2^d^  (40.9, 53.5) |

^1^Zinc deficiency is defined as a PZC <59 µg/dL in afternoon or < 66 µg/dL in morning (non-fasting). Prevalence estimates in the same row without the same superscript are statistically significant different from one another (p<0.05, adjusted using Bonferroni correction) using PROC GLIMMIX.

^2^alpha-1-acid glycoprotein (AGP); C-reactive protein (CRP); plasma or serum zinc concentration (PZC); regression correction (RC)

**Supplementary Table 5. Estimated prevalence of zinc deficiency after excluding subjects with inflammation in preschool children**^1,2^

|  | **Unadjusted** | | **Excluding observations where CRP > 5 mg/L** | | **Excluding observations where AGP > 1 g/L** | | **Excluding observations where CRP > 5 mg/L or AGP > 1 g/L** | |  |
| --- | --- | --- | --- | --- | --- | --- | --- | --- | --- |
| **Survey** | **N** | **% (95% CI)** | **N** | **% (95% CI)** | **N** | **% (95% CI)** | **N** | **% (95% CI)** |  |
| **Preschool children** | | | | | | | | | |
| Afghanistan | 658 | 25.5 (20.6, 30.3) | 595 | 23.7 (19.0-28.4) | 492 | 23.6 (18.6, 28.5) | 479 | 23.3 (18.4, 28.2) |  |
| Azerbaijan | 1016 | 14.0 (11.5, 16.4) | 933 | 13.3 (10.7-15.8) | 741 | 12.3 (9.6, 15.0) | 727 | 12.0 (9.3, 14.8) |  |
| Cambodia | 534 | 68.3 (61.0, 75.6) | 479 | 66.0 (58.7-73.4) | 305 | 64.0 (56.0, 72.0) | 297 | 63.0 (55.0, 71.0) |  |
| Cameroon | 776 | 80.0 (76.7, 83.3) | 493 | 76.1 (71.9-80.4) | 486 | 75.8 (71.4, 80.1) | 411 | 74.6 (70.0, 79.2) |  |
| Ecuador | 2017 | 27.7 (24.9, 30.5) | 1772 | 26.6 (23.2-30.0) | --- | --- | --- | --- |  |
| Malawi | 1071 | 61.3 (55.2, 67.4) | 845 | 60.4 (53.7-67.1) | 456 | 61.2 (52.5, 70.0) | 442 | 61.3 (52.4, 70.2) |  |

^1^Zinc deficiency is defined as a plasma or serum zinc concentration <57 µg/dL in afternoon or <65 µg/dL in the morning (non-fasting) in preschool children

^2^alpha-1-acid glycoprotein (AGP); C-reactive protein (CRP)

**Supplementary Table 6. Weighted Spearman correlation coefficients between PZC and CRP in preschool children and non-pregnant women of reproductive age after replacing CRP values below the limit of detection of 0.5 mg/L with a value of 0.25 mg/L**

|  | **PZC * CRP** | | |
| --- | --- | --- | --- |
| **Survey, year** | **N** | **r** | **p** |
| **Preschool children** |  |  |  |
| Azerbaijan, 2013 | 1016 | -0.06 | 0.08 |
| Cambodia, 2014 | 534 | -0.16 | 0.002 |
| Cameroon, 2009 | 776 | -0.24 | <0.0001 |
| Malawi, 2016 | 1071 | -0.12 | 0.01 |
| **Women of reproductive age** |  |  |  |
| Cambodia, 2014 | 693 | -0.03 | 0.44 |
| Cameroon, 2009 | 746 | -0.19 | <0.0001 |
| Malawi, 2016 | 760 | -0.02 | 0.66 |
